# Unconstrained image reconstruction with resolution modelling does not have a unique solution

**DOI:** 10.1186/s40658-014-0098-4

**Published:** 2014-11-30

**Authors:** Johan Nuyts

**Affiliations:** Department of Imaging and Pathology, Nuclear Medicine & Molecular imaging, Medical Imaging Research Center (MIRC), KU Leuven-University of Leuven, Herestraat 49, Leuven, B3000 Belgium

**Keywords:** PET, Resolution model, Gibbs artefact, Reconstruction

## Abstract

Positron emission tomography systems have a finite spatial resolution. When the system point spread function (PSF) is taken into account, the unconstrained reconstruction problem does not have a unique solution. As a result, the solution obtained with the maximum likelihood reconstruction algorithm typically suffers from Gibbs artefacts, which can have an adverse effect on tracer uptake quantification in small lesions. To deal with this problem, some assumptions about the undetected image features have to be introduced, either implicitly or explicitly. If one is willing to sacrifice resolution, the improvement of the PSF model can be used to suppress noise and at the same time impose a predefined (suboptimal) spatial resolution, facilitating pooled analysis of multicentre data.

**Electronic supplementary material:**

The online version of this article (doi:10.1186/s40658-014-0098-4) contains supplementary material, which is available to authorized users.

## Background

In nuclear medicine imaging, maximum-likelihood reconstruction is the preferred reconstruction algorithm, because it is relatively easy to program, it allows for a statistically correct treatment of the data and is flexible enough to enable an accurate modelling of the acquisition geometry and physics. Many researchers have reported that modelling the finite spatial resolution during iterative reconstruction results in ‘superior image quality’, where image quality may refer to various imaging tasks including lesion detection and tracer uptake quantification. Recently, the effect of resolution modelling on tracer uptake quantification in small lesions has been carefully investigated by several groups, because this is obviously very relevant for imaging in oncology. A study investigating the impact of resolution modelling on ^18^F-FDG-uptake in lung lesions appears in this issue [1]. The authors find that resolution modelling leads to an overestimation of the activity in small lesions, and they propose to post-smooth the image to improve quantification. This finding is somewhat counter-intuitive, since post-smoothing degrades the resolution, while improving it was the original motivation for introducing the resolution model. The authors offer an explanation for their finding. The aim of this commentary is to further clarify this finding and briefly discuss possible approaches to deal with this quantification problem.

## Main text

### The image of a point source

**Figure 1:**
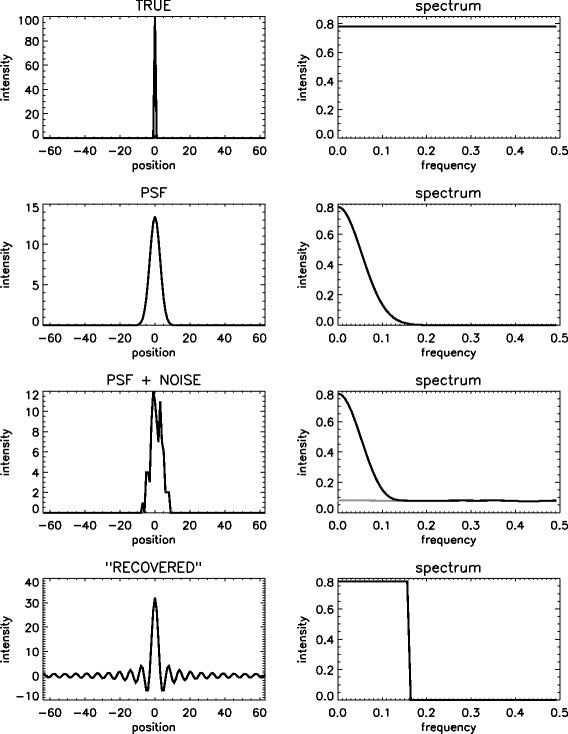
**Image and spectrum of a point source.** Top row: the ideal image of a point source and its frequency spectrum. Second row: noise-free blurred image of the point source and the corresponding frequency spectrum. Third row: blurred and (Poisson) noisy image of the point source and the corresponding frequency spectrum. The gray line is the spectrum of the noise, and the black line is the spectrum of the noisy image of the point source. Last row: The result of a (hypothetical) recovery procedure, restoring all non-zero frequency amplitudes.

The effects of blurring and deblurring are illustrated in Figure 1 using a simple 1D phantom, which is imaged by an idealised 1D detector. The first row shows the ideal image of a point source, and the corresponding frequency spectrum. All spatial frequencies are required for an accurate representation of a point source.

If this point source was imaged using a detector with a Gaussian point spread function, the image shown in the second row of the figure would be obtained. The corresponding frequency spectrum is shown as well. It reveals that the detector has good performance at low frequencies, while its performance decreases for increasing spatial frequencies. Near a frequency of 0.15 the detector performance decreases to zero. Because a Gaussian PSF was assumed, the frequency spectrum is Gaussian as well. In this plot, 0.5 is the Nyquist frequency.

If the image is produced by a short scan of a radioactive point source, it would be subject to Poisson noise as illustrated in the third row of Figure 1. Poisson noise is ‘white’, meaning that the expectation of its frequency spectrum is uniform. Thus, the noise basically adds a uniform background to the frequency spectrum.

Assume that a very performant deconvolution algorithm was applied to the image of the second row. This algorithm accurately recovers the amplitude of all frequencies that contributed to the data. Of course, the algorithm cannot recover frequency amplitudes that were set to zero by detector blurring. The resulting frequency spectrum has the correct amplitude for all frequencies up to about 0.15. The amplitude of higher frequencies remains zero because there was no signal to recover. The image that is produced by this “optimal” recovery of the available frequencies is shown on the left in the last row. It has a peak which is significantly narrower and higher than that of the original Gaussian, indicating that the spatial resolution of the system was improved as intended. Unfortunately, this narrow peak is surrounded by oscillating ‘tails’, also known as ringing. This function is the so-called sinc function, here defined as

1 $$\text{sinc}(\text{ax})=\frac{a}{\pi }\;\frac{\sin(\text{ax})}{\text{ax}},$$

where *x* is the position and *a* determines the width of the function. Because the system cannot detect the higher frequencies, it is unable to detect the difference between activity concentrated in a single point source (first row of Figure 1) and the same amount of activity distributed as a sinc function (fourth row).

### The Gibbs artefact

The sinc appears as the ‘natural’ PSF when one deconvolves for known blurring, using the assumption that details which could not be detected should be ignored. Unfortunately, the sinc has two features which can be undesirable for particular imaging tasks. These features are 1) the presence of decaying, oscillating tails, which can be disturbing for visual analysis and 2) the fact that the integral of the central part of the sinc (1) is larger than one:

2 $$\begin{array}{ccc} \overset{\infty }{\underset{-\infty }{\int }}\frac{a}{\pi }\;\frac{\sin(\text{ax})}{\text{ax}}\text{dx} & = & 1 \end{array}$$

3 $$\begin{array}{ccc} \overset{\pi /a}{\underset{-\pi /a}{\int }}\frac{a}{\pi }\;\frac{\sin(\text{ax})}{\text{ax}}\text{dx} & ≃ & 1.18 \end{array}$$

The interval [−*π*/*a*,*π*/*a*] covers the central nonnegative part of the sinc. Since the integral of this central part is larger than one, the two tails are negative, each with a value of about −0.09.

**Figure 2:**
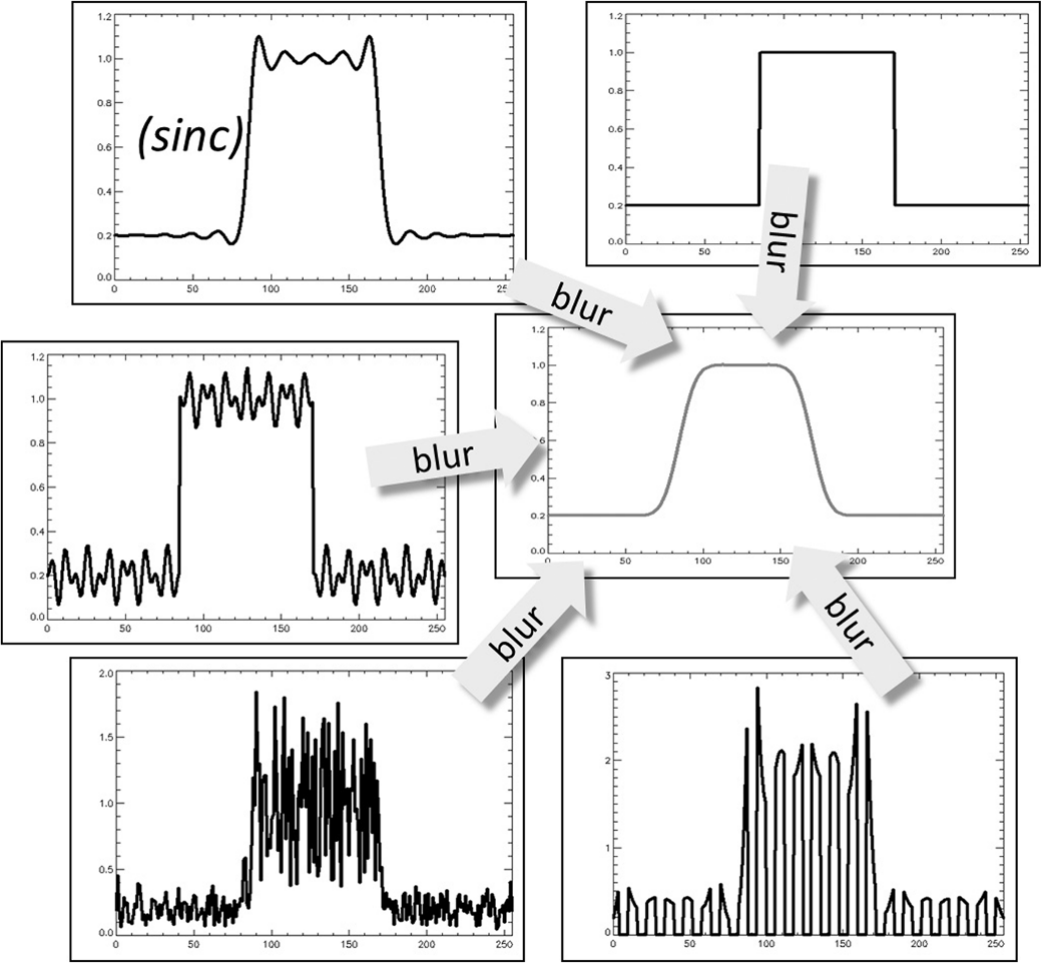
**Different solutions to the deconvolution problem.** All these profiles produce the same blurred block profile after Gaussian smoothing. The image marked ‘sinc’ is the solution obtained by setting all undetected spatial frequencies to zero. The other images have non-zero contributions from some or all of these frequencies.

Suppose we have a hot uniform 1D object in a low activity background, as illustrated in the top right plot of Figure 2. If one convolves this function with a sinc, the activity near the edges in the object is amplified with a factor of about 1.09. This is because during convolution, the center and one tail cover the uniform object, producing an activity of 1.09, while the other tail covers the background, and therefore contributes little to the convolution. The convolution of this uniform object with a sinc is shown in the top left plot of Figure 2 (marked ‘sinc’). This is the image obtained when one deblurs an image with the ‘optimal’ deconvolution algorithm described previously.

If the uniform object were smaller, exactly matching the central part of the sinc, then the convolution with the sinc would produce its maximum amplification of approximately 1.18. Consequently, deblurring objects with a size similar to the system PSF can create a significant overestimation of the activity. This simplified case only considered a single dimension, in multiple dimensions the effects in each dimension are combined, which may produce an even higher amplification (reaching a maximum for a cuboid object, which is fortunately not a typical shape in biological systems).

As mentioned before, the Gibbs artefact is created by restoring the amplitude of the detected frequencies, while setting the amplitudes of undetectable frequencies to zero. Since these frequencies are not detected by the system, any amplitude can be assigned to them. Each different choice produces a different solution to the deblurring problem. This is illustrated in Figure 2, where different 1D objects are shown which produce exactly the same result after blurring with the system PSF.

**Figure 3:**
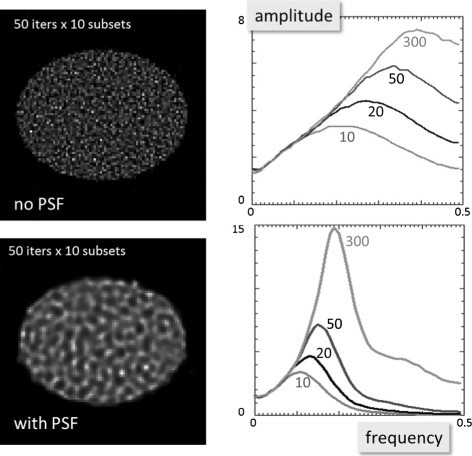
**Noisy reconstructions and noise power spectrum.** The images are OSEM reconstructions of a uniform disk from noisy projections ignoring the PSF (top row) and using a PSF model (bottom row). The plots show the corresponding noise power spectra for different iteration numbers (10, 20, 50, 300). The noise power increases with increasing interation number.

For PET reconstruction, the ordered-subsets expectation-maximisation (OSEM) algorithm is typically used. Because OSEM with PSF modelling applies the PSF blurring in both the forward and the back projections, it has a strong tendency to assign a zero amplitude to undetectable frequencies. Consequently, OSEM reconstructions tend to deblur the Gaussian PSF into a narrower sinc-like PSF. On the other hand, zeroing the amplitudes of higher frequencies results in strong noise suppression, so the resulting images not only look sharper, they are also less noisy. This is illustrated in Figure 3, showing the OSEM reconstructions of a uniform disk from a (simulated) noisy 2D sinogram. The figure also shows plots of the noise spectra for different iteration numbers. If the PSF is not modelled, then applying more iterations increases the noise in the higher frequencies, making the noise proportional to the frequency after many iterations. In contrast, when the PSF is modelled, the noise at higher frequencies is strongly suppressed. Additional iterations mostly increase mid-frequency noise. This produces a different noise pattern, which could be misleading for readers who are unfamiliar with this type of reconstruction. An interesting discussion on this issue between Alessio and Rahmim can be found in [2].

Note that since OSEM combines deblurring with reconstruction in a maximum-likelihood framework, its deblurring performance is somewhat different and more complicated than that of the simplistic 1D deblurring operator discussed above [3],[4].

### Post-smoothing, blobs and priors

To suppress the Gibbs artefacts, the steep cutoff in the frequency spectrum of the object must be avoided. One approach is to give up on resolution recovery, and assume that the object does not consist of a collection of point sources, but of a collection of blobs, where the blob is designed to be similar to the PSF. This can be achieved in several ways. One approach, similar to the one discussed in [1], is to model the PSF during reconstruction and post-smooth the final image with a kernel approximating the PSF. This post-smoothing obviously degrades the resolution again, making it similar to what would have been obtained with OSEM without resolution model and without post-smoothing. However, the post-smoothing suppresses the noise in the higher frequencies. A very similar effect can be obtained by incorporating a PSF-like blob directly into the reconstruction (and not including the PSF in the system matrix). Instead of modelling the image as a combination of point sources, it is modelled as a combination of Gaussian blobs (also called sieves) [5] or Kaiser-Bessel functions [6]. The noise in the higher frequencies (see Figure 1, row 3) cannot be represented as a combination of these blobs and is therefore not incorporated in the reconstruction, resulting in significant noise suppression. A third but more complex approach is to use a penalised-maximum likelihood algorithm [7] with a penalty designed to impose a predefined target impulse response [8]. With all these approaches, the knowledge of the system PSF is used to suppress noise, sacrificing some or all of the gain in resolution that could have been obtained. An interesting side effect of these approaches is that they can produce images with a fairly shift invariant, isotropic and predictable spatial resolution (provided that a sufficient amount of iterations is applied). In contrast, OSEM with a low number of iterations or standard penalised likelihood approaches produce images with a non-uniform spatial resolution. The reconstruction of images with known and approximately shift invariant resolution is very important when pooling images from different PET systems in clinical trials [1].

Another approach to suppress the Gibbs artefacts is to use penalties in a penalised likelihood reconstruction algorithm, or equivalently, priors in a maximum *a posteriori* reconstruction algorithm. Usually convex penalties are used, which ensure that the problem has a unique solution. When such a penalty is applied to the problem of Figure 2, it would take a different value for all the possible solutions to the unconstrained problem. For this particular 1D problem, the best penalty would be one that favours piecewise smooth solutions. An example of penalty design for clinical PET reconstruction is [9]. Even better penalties can be designed when additional knowledge about the patient anatomy is available from other imaging modalities such as CT or MR. These penalties typically assume that anatomical boundaries tend to coincide with metabolic boundaries, and therefore avoid smoothing over anatomical boundaries. Since the anatomical images usually have a better resolution than the PET system, this additional knowledge helps to assign meaningful amplitudes to detail that was invisible to the PET system [10]. This approach can produce images with excellent visual and quantitative properties [11]. However, they have a very non-uniform spatial resolution and training is required to make optimal use of the diagnostic information in these images.

## Conclusion

When the finite spatial resolution of the PET system is taken into account during reconstruction, ‘better’ images should be obtained because more knowledge about the acquisition system was used. Indeed, many researchers have reported that the images not only become sharper, they also tend to be less noisy. Unfortunately, taking the resolution into account also makes the reconstruction problem underdetermined: different images exist that agree equally well with the acquired data. The maximum-likelihood (or OSEM) reconstruction algorithm tends to select a solution suffering from Gibbs artefacts. This problem can be mitigated by post-smoothing the OSEM images or by using penalised likelihood reconstruction algorithms with a penalty that favours smoothness. With these approaches, the gain obtained by using a better model is not (or not entirely) used to improve the resolution, but instead to suppress noise and by doing so, avoid the introduction of Gibbs artefacts. For analysis of multicenter data the amount of smoothing can be selected such that the images from all the PET systems involved are reconstructed with very similar spatial resolution.

## Authors’ original submitted files for images

Below are the links to the authors’ original submitted files for images. Authors’ original file for figure 1 Authors’ original file for figure 2 Authors’ original file for figure 3
