# Supplementary figures and images for: Unconstrained image reconstruction with resolution modelling does not have a unique solution
Source: EJNMMI Phys. 2014 Nov 30;1:98. doi: 10.1186/s40658-014-0098-4 (PMC4545809; doi:10.1186/s40658-014-0098-4)

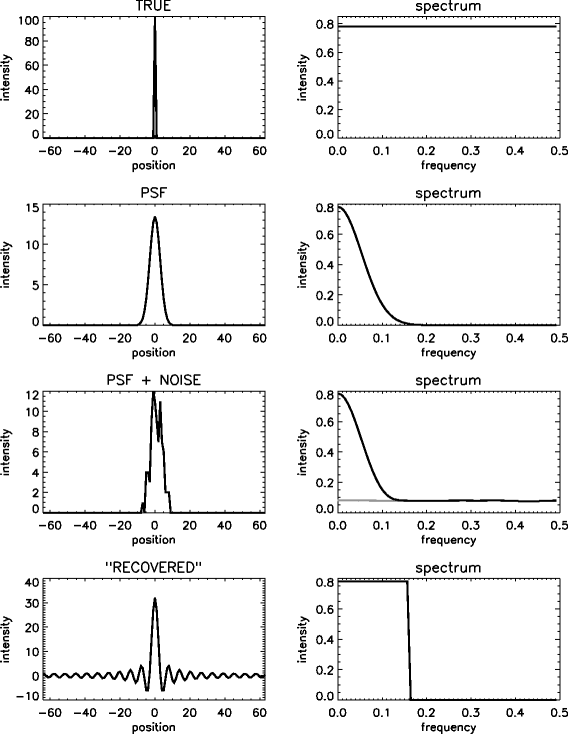

Supplement: Supplementary file 1 — Authors’ original file for figure 1 [file 40658_2014_98_MOESM1_ESM.gif]

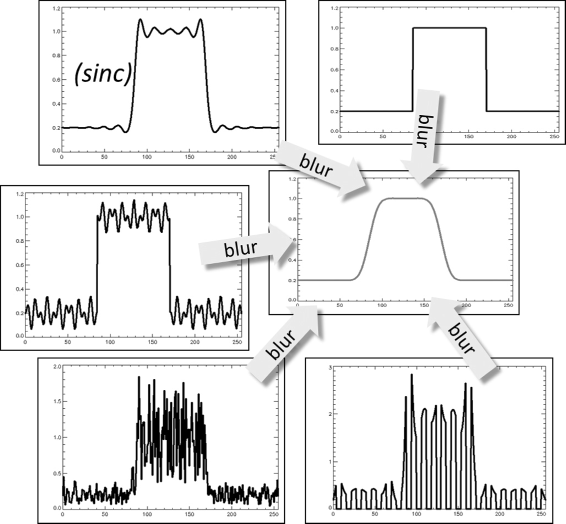

Supplement: Supplementary file 2 — Authors’ original file for figure 2 [file 40658_2014_98_MOESM2_ESM.gif]

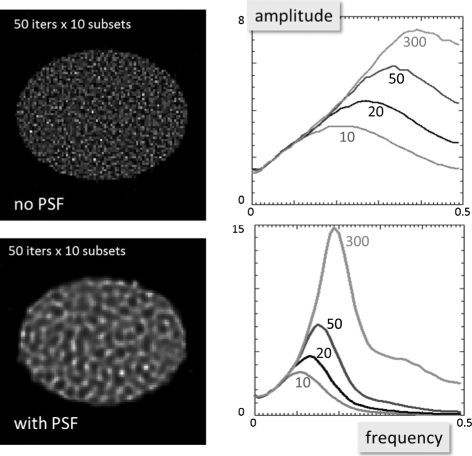

Supplement: Supplementary file 3 — Authors’ original file for figure 3 [file 40658_2014_98_MOESM3_ESM.gif]
